# Screening of sugarcane germplasm against *Sporisorium scitamineum* and its effects on setts germination and tillering

**DOI:** 10.1038/s41598-024-64810-1

**Published:** 2024-06-25

**Authors:** Muhammad Aslam Rajput, Owais Iqbal, Rehana Naz Syed, Heba H. Elsalahy, Nasir Ahmed Rajput, Sagheer Ahmad, Rizwan Khan, Muhammad Ali Khanzada, Muhammad Usama Younas, Muhammad Qasim, Humaira Rizwana, Khalid S. Almaary, Rashid Iqbal, Abdul Mubeen Lodhi

**Affiliations:** 1 CDRI, Pakistan Agricultural Research Council, University of Karachi, Karachi, 75270 Pakistan; 2 Department of Plant Protection, Faculty of Crop Protection, Sindh Agriculture University, Tandojam, 70060 Pakistan; 3 State Key Laboratory for Conservation and Utilization of Bio-Resources in Yunnan, Yunnan Agricultural University, Kunming, 650201 China; 4 Leibniz Centre for Agricultural Landscape Research (ZALF), 15374 Müncheberg, Germany; 5 Department of Plant Pathology, University of Agriculture, Faisalabad, 38000 Pakistan; 6 Plant Sciences Division, Pakistan Agricultural Research Council, Sector G-5/1, Islamabad, Pakistan; 7 Key Laboratory of Plant Functional Genomics of the Ministry of Education/Jiangsu Key Laboratory of Crop Genomics and Molecular Breeding, Agricultural College of Yangzhou University, Yangzhou, China; 8 Microelement Research Center, College of Resources and Environment, Huazhong Agricultural University, Wuhan, 430070 Hubei China; 9 Department of Botany and Microbiology, College of Science, King Saud University, P.O. 2455, 11451 Riyadh, Saudi Arabia; 10 Department of Agronomy, Faculty of Agriculture and Environment, The Islamia University of Bahawalpur, Bahawalpur, 63100 Pakistan

**Keywords:** Sugarcane, Host response, Germination, Tillering, Whip smut, Resistance, Genetics, Plant sciences

## Abstract

Sugarcane smut is the most damaging disease that is present almost across the globe, causing mild to severe yield losses depending upon the cultivar types, pathogen races and climatic conditions. Cultivation of smut-resistant cultivars is the most feasible and economical option to mitigate its damages. Previous investigations revealed that there is a scarcity of information on early detection and effective strategies to suppress etiological agents of smut disease due to the characteristics overlapping within species complexes. In this study, 104 sugarcane cultivars were screened by artificial inoculation with homogenate of all possible pathogen races of *Sporisorium scitamineum* during two consecutive growing seasons. The logistic smut growth pattern and the disease intrinsic rate were recorded by disease growth curve. Variable levels of disease incidence i.e., ranging from 0 to 54.10% were observed among these sugarcane cultivars. Besides, pathogen DNA in plant shoots of all the cultivars was successfully amplified by PCR method using smut-specific primers except 26 cultivars which showed an immune reaction in the field trial. Furthermore, the plant germination and tillering of susceptible sugarcane cultivars were greatly influenced by pathogen inoculation. In susceptible cultivars, *S. scitamineum* caused a significant reduction in setts germination, coupled with profuse tillering, resulting in fewer millable canes. Correlation analysis demonstrated that there was a positive relationship between reduction in setts germination and increase in the number of tillers. The present study would be helpful for the evaluation of smut resistance in a wide range of sugarcane germplasm, especially from the aspects of setts germination and tillers formation, and it also screened out several excellent germplasm for potential application in sugarcane breeding.

## Introduction

Sugarcane smut is caused by the dimorphic basidiomycetous fungus *Sporisorium scitamineum* (Sydow) M. Piepenbr., M. Stoll & Oberw. (Syn: *Ustilago scitaminea* H. & P. Sydow). It was first reported in Natal (South Africa) in 1877^1^. Since then, it became prevalent in almost all cane-growing regions of the world except Papua New Guinea and Fiji^2–5^. However, sugarcane smut has been found in Papua New Guinea in 2016^6^. It is characterized by the emergence of a typical structure, called ‘smut whip’^1^. The infected sugarcane plants are usually stunted in growth and produce thin slender canes generally with broad-spaced nodes, with a whip-like sorus^2^. Generally, the infected sugarcane plants produce profuse tillers with the shoots being spindlier and erect with smaller narrow leaves^7^. The affected plants are severely stunted and yield losses may range from 12–75%. Total crop failure may be possible, if susceptible cultivars are grown and climatic conditions are favorable for infection^7^. In the past, several superior cultivars have been eliminated for cultivation due to this disease^8–10^. Cultivation of resistant cultivars is the most reliable and practicable control measure to minimize the adverse impact of this disease. Previous studies have revealed that polygenic resistant towards this disease is present in sugarcane cultivars as suffered plants show different types of reaction^11^. Sugarcane genes resistant to whip smut pathogen have been tagged^12^. It is demonstrated that genetic diversity exists within the whip smut pathogen population^13,14^, and that also applied to the smut pathogen of Pakistan^15^. Abundant pathogen races make the resistant screening and disease management more complicated. Through the detection of pathogen proliferation and the determination of physiological index, pathogen may be detected even earlier than the symptoms expression^16^. PCR-based methods and protein-based techniques were used to tag the corresponding gene/protein in the host^17^. Many workers successfully amplified and visualized *S. scitamineum* DNA present in the plant tissues^18^.

Whip smut is one of the most widely distributed diseases in almost all cane growing areas, thereby causing substantial losses. This disease is unbearable for the countries like Pakistan, which mainly depends on sugarcane for sugar production. Therefore, we evaluated a wide range of sugarcane germplasm against this disease and measure its effects on germination and tillers formation, and also aimed to screen out several excellent germplasm for potential application in sugarcane breeding.

## Materials and methods

### Source of cultivars, experimental site and design

A total of 104 sugarcane cultivars were collected from various sources and maintained at National Sugar and Tropical Horticulture and Research Institute (NSTHRI), Thatta (Supplementary table 1). The experiment was conducted (experimental studies and experimental materials involved in this research are in full compliance with relevant institutional, national and international guidelines and legislation) at Agriculture Research Institute, Tandojam, Pakistan (250 25.19 N; 680 32.07 E). Sugarcane setts were obtained from the 12-month-old plants that were healthy and free from whip smut disease. To eliminate the chances of natural infection, all setts were treated in hot water at 52 °C for 30 min before further use. Buds were exposed by removing the leaves and arranged as three buds per sett, sown in the 5-m ridges with the row-to-row distance of one meter. The experiment was carried out in the soil which had no previous history of plantation of sugarcane crop and arranged in a split-plot design. This was conducted to consider that the soil was free from *S. scitamineum* and also to avoid the cross-contamination. This study was conducted for two consecutive seasons (2014–15 and 2015–16). Cultivars were grown with 6 replications in which disease treatments (inoculated & un-inoculated) served as whole plots (Factor A) and cultivars as sub-plots (Factor B) randomized within each block. Meteorological data regarding temperature, moisture and rainfall were obtained from the Meteorological Station of Agriculture Research Institute Tandojam.

### Pathogen collection and inoculation

Newly developed whips were collected in paper bags from all cane growing areas of Sindh province to ensure that collected inoculum comprised all possible pathogen races. These whips were shade dried at room temperature; teliospores were harvested, packed in cellophane bags and stored at 4 °C. At the time of planting, the viability of these teliospores was checked on 1.5% water agar, teliospores having at least 90% germination were used for inoculating the setts^19^. The teliospores suspension was prepared by adding 25 g of viable smut teliospores into 50 L of distilled sterilized water along with Tween-20 should be added proportionally. The final concentration of 5 × 10^6^ teliospores/ml was maintained with the help of a hemocytometer^20^. The healthy, hot water-treated 3 budded setts were dipped into the prepared spore suspension for 30 min and kept in polythene bags overnight before planting^21^. Setts dipped in only distilled sterilized water served as control (un-inoculated).

### Germination, tillering and disease intensity

Setts germination was recorded up to 60 days of sowing. The collected data was transformed into percent germination by using the formula:

$${\text{Germination}}\;\left( \% \right) = \left( {{\text{Number}}\;{\text{of}}\;{\text{buds}}\;{\text{germinated/Total}}\;{\text{number}}\;{\text{of}}\;{\text{buds}}\;{\text{ planted}}} \right) \times {1}00\%$$

The number of tillers originated from each plant (bud) was counted till 120 days of the plantation. Smut incidence was recorded on monthly basis after 3 months of the plantation. Smut clumps and whips appeared were counted and roughed out after each observation and destroyed to avoid secondary infestation. The disease severity was determined by using 0–9 disease rating scale^22^ (Table 1), and the disease incidence was computed by using the following formula:

$${\text{Disease}}\;{\text{incidence}}\;\left( \% \right) = \left( {{\text{Number}}\;{\text{of}}\;{\text{infected}}\;{\text{stools/Total}}\;{\text{number}}\;{\text{of}}\;{\text{stools}}} \right) \times {1}00\%$$

**Table 1:** A whip smut disease rating scale ranging from 0 to 9.

Disease incidence (%)	Disease rating	Reaction group
0	0	Immune
0.1–2.5	1	Very highly resistant (VHR)
2.6–5.5	2	Highly resistant (HR)
5.6–7.5	3	Resistant (R)
7.6–2.5	4	Moderately resistant (MR)
12.6–15.5	5	Intermediate (I)
15.6–18.0	6	Moderately susceptible (MS)
18.1–22.5	7	Susceptible (S)
22.6–25.5	8	Highly susceptible (HS)
25.6–100	9	Very highly susceptible (VHS)

### Cultivars screening by PCR against smut infection

DNA of all cultivars which were inoculated at the time of planting with a viable inoculum of *S. scitamineum* as described above were extracted and amplified to confirm the presence or absence of smut pathogen within the host tissues. For this purpose, after two months of planting, top shoots of nine randomly selected plants (three from each row) of each cultivar were collected for DNA extraction, by using slightly modified MATAB (mixed alkyl trimethyl-ammonium bromide) method^23,24^. In present experiment, after placing of plant material in MATAB solution and subsequent heating and cooling, we also added 10μl of RNase to obtained better quality of DNA. The centrifuge tubes then vortexed for 20 s and incubated at 37°C for 30 min, then cool down the tubes by placing on ice for 30 min. All other procedure was same as described by Moosawi-Jorf et al. and Bibi et al.^23,24^. The resulting DNA was quantified with the help of a spectrophotometer (BIOMATE-3, Thermo Fisher Sci, USA) at absorbance 260/280 nm.

DNA was amplified by using smut-specific forward and reverse primers i.e., (S1) 3-GCAGCCGATAATCTACCAA-5 and (S2) 5-CCAGCTTCTTGCTCATCCTC-3^25^. PCR reaction was carried out in 10μl reaction mixture containing 25ng of the template (Genomic DNA), 0.2 mM of each dNTPs, 0.5 mM MgCl, 0.1 U of Taq polymerase (Eppendorf, Hamburg, Germany), 0.10 μM of Reverse primer and 0.08 μM of forwarding primer in a 1X PCR reaction buffer. The amplification reaction was performed in the Eppendorf Master Cycler (6332, Nexus Eco Thermal cycler) with an initial denaturation for 5 min at 94°C, then 35 cycles: 30 s denaturation at 94°C; 30 s annealing at 56°C; 60 s extension at 72°C. The final extension was carried out at 72°C for 5 min. Amplified products were analyzed through electrophoresis (HU-20, UK) on 2% agarose (Applichem, Germany) gel containing 0.5X TBE (Tris Borate EDTA) at 72 V for 2 h and stained with ethidium bromide (0.5μg/ml) (Sigma Aldrich, USA). The resulting bands of 450 bp were visualized under UV light (260nm) using a gel documentation system (Vilber Lourmat, France).

### Data analysis

Statistical parameters such as mean, standard deviation, analysis of variance, LSD multiple comparison tests, paired t-test, and regression equations were calculated using the Statisix-8.1 package.

## Results

### Response of sugarcane cultivars to *S. scitamineum*

**Figure 1:**
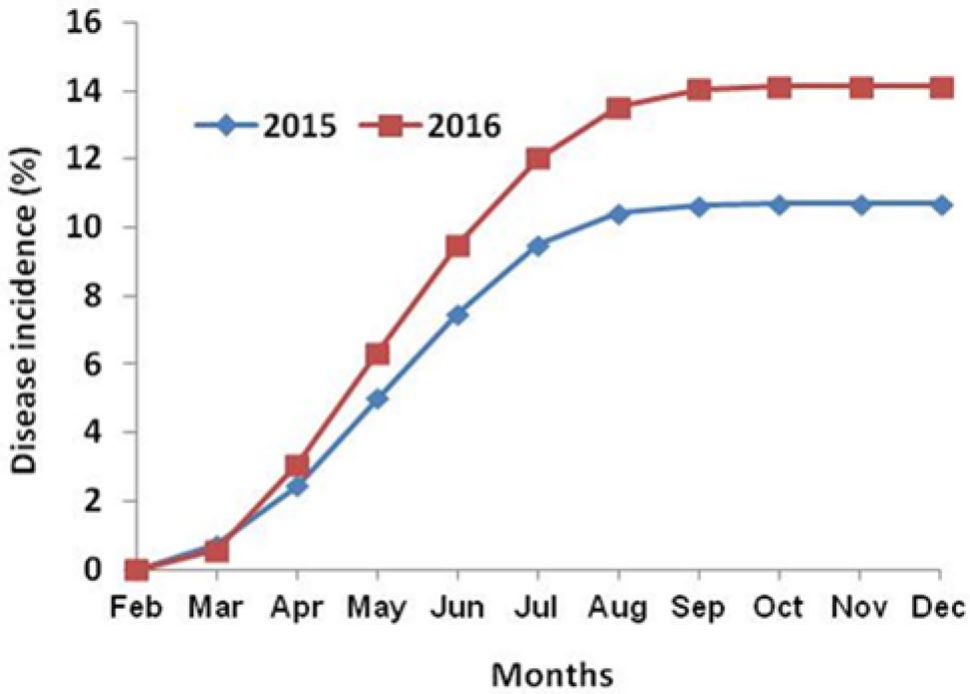
Incidence of sugarcane smut during two cropping seasons. Disease incidence of all the tested cultivars were pooled for each time point.

The results of 104 sugarcane cultivars that were artificially inoculated with *S. scitamineum* in the field screening trials during two consecutive seasons were rated on 0–9 scale (Table 1). The disease growth curve (based on the average of all the cultivars tested) with time in months for both copping years indicated the logistic smut growth pattern. The whip smut intensity was more in 2016 as compared to 2015 on tested sugarcane cultivars. At the initial crop growth stage, the disease progression was slow, but an exponential increase in disease development occurred during April to August in both growing seasons. From August to December, the whip smut disease in transitional to the stationary phase means no noticeable increase in disease development was noticed during this period (Fig. 1). As disease intrinsic rate was recorded in September, thus it is used to compute ultimate disease incidence as well as for disease rating and host response.

The cultivars were classified into different groups according to their reaction type. The results showed that out of 104 sugarcane cultivars tested, 26 cultivars, namely S-2006-SP-30, BPTh-807, CP-70–530, S-2003-QSSG-776, S-2003-US-633, QSG-1741, HoTh-516, HoTh-438, CB-2919, HoTh-544, Th-704, HoTh-318, HoTh-610, HoTh-4140, Roc-16, AP-98–156/02, AP-98–156/03, AP-98–156/04, AP-04–68/01, AP-98–156/07, AP-98–103/01, AP-97–69/01, AP-97–56/02, AP-97–56/03, BP-TJ-651/18 and BP-TJ-651/20 were found immune (Supplementary table 2).

The other 78 cultivars in the trial showed different levels of smut incidence and were characterized in different groups; 2 cultivars, HoTh-344 and AP-04–46/03 with 1.53 and 2.31% disease incidence were ranked as ‘Very Highly Resistant’ (VHR), 4 were marked as ‘Highly Resistant’ (HR). Fifteen were characterized as ‘Resistant’ (R), 27 were found ‘Moderately Resistant’ (MR), 8 were ‘Intermediate’ (I), 6 were found ‘Moderately Susceptible’ (MS), 10 were ranked as ‘Susceptible’ (S), 3 were found ‘Highly susceptible’ (HS) and 3 namely CSSG-1741, HoTh-550 and CP-29–120 were marked as ‘Very Highly Susceptible’ (VHS). The results revealed that the maximum disease incidence was developed in CP-29–120 (54.10%), followed by Hoth-550 (33.76%), CSSG-1741 (29.48%) and Co-208 (25.43%); while the minimum disease incidence was observed in Hoth-344 (1.53%), followed by AP-04–46/03, Hoth-518 (3.27%) and CP-82–2083 (4.28%) (Supplementary table 2).

### Molecular diagnosis of whip smut *S. scitamineum* from sugarcane shoots

For accurate identification of pathogen presence in sugarcane cultivars and comparison of morphological diagnosis method, DNA was extracted from each used cultivar. For this purpose, DNA was extracted from pathogen-inoculated cultivars and amplified by using smut-specific primers (Supplementary Fig. 1). Out of 104 entries, only 78 have successfully amplified giving bands of 450 bp. While, the rest of samples did not produce any amplification and have no band. These results are according to the morphological disease scoring method (Supplementary table 2).

### Impact of environment on whip smut development

The Pearson correlation between environmental factors and disease incidence percentage is presented in (Table 2). It demonstrated that both minimum and maximum temperatures were differently linked with disease development. The minimum temperature was positively correlated with the disease incidence percentage in 2014–15 and overall, but negatively in 2015–16 cropping season. The result further indicated that there was no significant effect of minimum temperature on disease progression in both years. Furthermore, the correlation between maximum temperature and smut incidence was negative and weak in both years as well as overall, but shows a significant influence on disease development. On the other hand, the correlation between disease incidence percentage and relative humidity was positive and strong in both cropping seasons and overall. The r values for 2014–15, 2015–16 and overall were r = 0.7581, r = 0.6290 and r = 0.7100, respectively. Data for the correlation between the rainfall during the period of study and the disease incidence revealed a positive, but very weak and non-significant relationship between both variables (Table 2).

**Table 2:** Correlation between environmental factors and disease incidence percentage recorded during study period.

Environmental factors	Disease incidence percentage		
	2014–15	2015–16	overall
Temperature (minimum)	r = 0.0443	r = − 0.0140	r = 0.0138
	0.9032^NS^	0.9694^NS^	0.9699^NS^
Temperature (maximum)	r = − 0.3257	r = − 0.2387	r = − 0.2828
	0.3584*	0.5066	0.4286*
Relative humidity	r = 0.7581S	r = 0.6290S	r = 0.7100S
	0.0110*	0.0514	0.0214*
Rain fall	r = 0.1463	r = 0.1571	r = 0.1504
	0.6867^NS^	0.6647^NS^	0.6784^NS^

The *P*-values with * are significant whereas NS means non-significant values.

### Effect of *S. scitamineum* on germination

The pathogen significantly reduced germination in most of the sugarcane cultivars tested. In terms of setts germination percentage, analysis of variance revealed a highly significant difference between inoculated (48.80%) and un-inoculated (58.01%) treatments. The cultivars main effect (DF = 103, F = 152.86, *P* = 0.000), pathogen treatment's main effect (DF = 1, F = 3629.25, *P* = 0.000) and cultivars × treatment's effect (DF = 103, F = 101.59, *P* = 0.000) were highly significant (Tables 3 and 4).

**Table 3:** Analysis of variance of cultivars and disease treatments and its association with setts germination.

Source	Degrees of freedom	Sum of squares	Mean sum of squares	F-Ratio
A (Treatments)	1	26,492.5	26,492.5	3629.25***
Block (Replicates)	5	19.2	3.8	
Error A (Block*Treatments)	5	36.5	7.3	
B (Cultivars)	103	14,587.0	141.6	152.86***
A*B (Treatments*Cultivars)	103	9694.3	94.1	101.59***
Error B (Block*Treatments*Cultivars)	1030	954.3	0.9	
Total	1247	51,783.8		

**** *P* < 0.0001.

**Table 4:** The main effects of disease treatments on sugarcane germination and tillering.

Parameters	Treatments		LSD
	Inoculated	Un-inoculated	
Germination	48.798^b^	58.013^a^	0.1087
Tillering	5.7692^a^	5.0808^b^	0.0434

The data is compared horizontally. The data with different letters are significantly different from one another.

Setts germination of all the 104 cultivars in field trials in both treatments as well as calculated reduction percentages and t-values are given in (Supplementary table 3). The artificial infestation of setts by smut pathogen had brought a highly significant reduction (t > 4.032), ranging from 10.38–26.08% in 78 cultivars. The most affected cultivars included CP-82–2083 (26.08%), Co-208 (25.96%), S-2006-SP-18 (25.66%) and AP-04–59/03 (25.48%). On the other hand, the pathogen failed to cause negative effects on germination of 26 inoculated cultivars, showing non-significant (t < 2.57) reduction. The results also revealed that there mostly was no significant difference in the germination of inoculated and un-inoculated cultivars, which appeared immune against whip smut infection. In pathogen inoculated setts, maximum mean germination was observed in HoTh-516 (66.22 ± 0.36%) followed by CPS-1827 (57.28 ± 0.49%), AP-98–156/05 (57.10 ± 0.67%) and AP-98–156/07 (56.78 ± 0.15%). While minimum germination in inoculated setts were recorded in Co-1148 (39.32 ± 0.28%) followed by CSSG-1741 (40.99 ± 0.25%), Hoth-550 (41.26 ± 0.33%) and Co-620 (41.62 ± 0.28%). Maximum germination in un-inoculated plots was recorded in AP-98–156/05 (69.92 ± 0.32%) followed by AP-98–156/06 (67.57 ± 0.20%), Q-88 (67.48 ± 0.21%) and HoTh-516 (66.27 ± 0.30%); while, minimum germination was occurred in S-2003-QSSG-776 (47.17 ± 0.72%) followed by Roc-16 (48.81 ± 0.17%), Hoth-550 (49.01 ± 0.29%) and Co-1148 (49.34 ± 0.26%) as shown in (Supplementary table 3).

**Figure 2:**
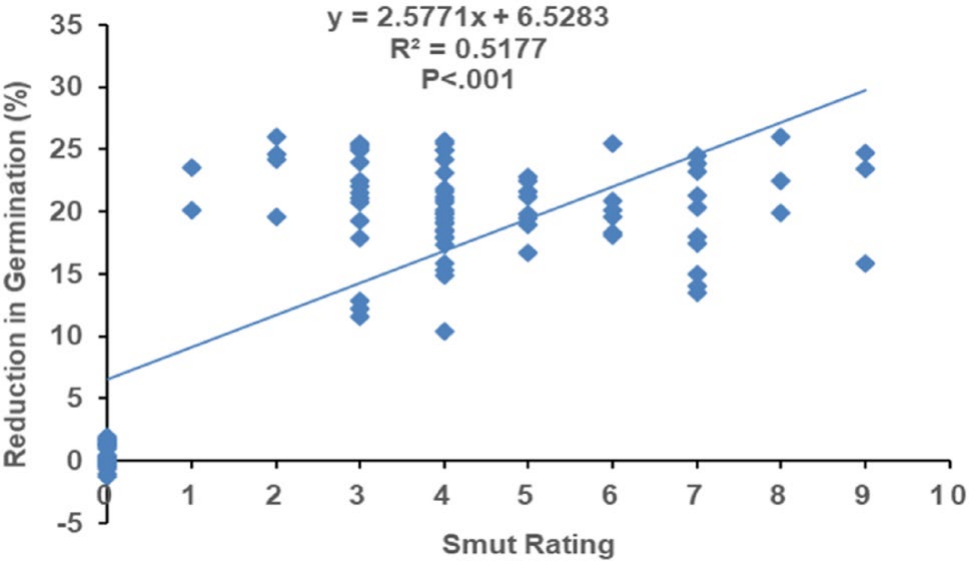
Regression analysis of plant germination with the whip smut disease rating. Data for plant germination of tested cultivars against each disease rating category was pooled and their means were used for regression analysis. Dots representing 104 cultivars artificially infested with *Sporisorium scitamineum*. Plant germination was recorded till 60 days of sowing.

The least-square linear regression analysis showed that the smut rating and reduction percentage of germination was moderately related to each other (R^2^ = 0.5177). The statistical analysis further indicated that there was a significant (*P* < 0.001) effect of the disease incidence on germination. Moreover, according to the regression equation (y = 2.57711x + 6.5283), it was observed that 2.58% germination at zero level of smut rating; further predicted that one unit change in smut rating can influenced 6.5283% setts germination (Fig. 2).

### Effect of *S. scitamineum* on tillers/plants

Significant variations were found in terms of the number of tillers/plants between inoculated and un-inoculated plots. On an overall basis, cultivars showed highly significant impact (DF = 103, F = 21.56, *P* = 0.000). Moreover, pathogen infection also adversely affected the tillering in susceptible cultivars. The pathogen treatment's effect was also highly significant (DF = 1, F = 163.91, *P* = 0.000). The interactive effect of cultivars and treatments also appeared highly significant (DF = 103, F = 10.31, *P* = 0.000) (Tables 4 and 5).

**Table 5:** Analysis of variance of cultivars and disease treatments associated with tillers/plant.

Source	DF	SS	MS	F-Ratio
A (Treatments)	1	147.840	147.840	163.91
Block (Replicates)	5	2.714	0.543	
Error A (Block*Treatments)	5	4.510	0.902	
B (Cultivars)	103	331.088	3.214	21.56***
A*B (Treatments*Cultivars)	103	158.382	1.538	10.31***
Error B (Block*Treatments*Cultivars)	1030	153.580	0.149	
Total	1247	798.114		

**** *P* < 0.0001.

In the case of a whip smut disease, the susceptible cultivars produced more tillers as compared to the resistant ones. Accordingly, we found no significant difference in tillers/plants, which showed an immune reaction. Based on tillers/plants, the tested cultivars can be divided into three groups. The first group comprises 44 cultivars of immune or highly resistant cultivars, showing non-significant (t < 2.571) differences between pathogen-inoculated and un-inoculated setts in terms of tillers/plant. The 2nd group consists of 26 cultivars, showing significant differences (t > 2.571) in tillers/plants between inoculated and un-inoculated setts. The inoculated cultivars of this group produced 6.92 to 12.81% more tillers. The 3rd group contains 34 cultivars, in which highly significant differences (t > 4.032) were recorded in tillers on inoculated and un-inoculated plants. This group of inoculated cultivars produced 13.58 to 34.86% more tillers as compared to the un-inoculated ones. Maximum increase in tillers/plant was recorded in CP-29–120 (34.86%), followed by CSSG-1741 (33.35%), Hoth-408 (33.32%) and CPD-01–359 (33.08%) (Supplementary table 4).

**Figure 3:**
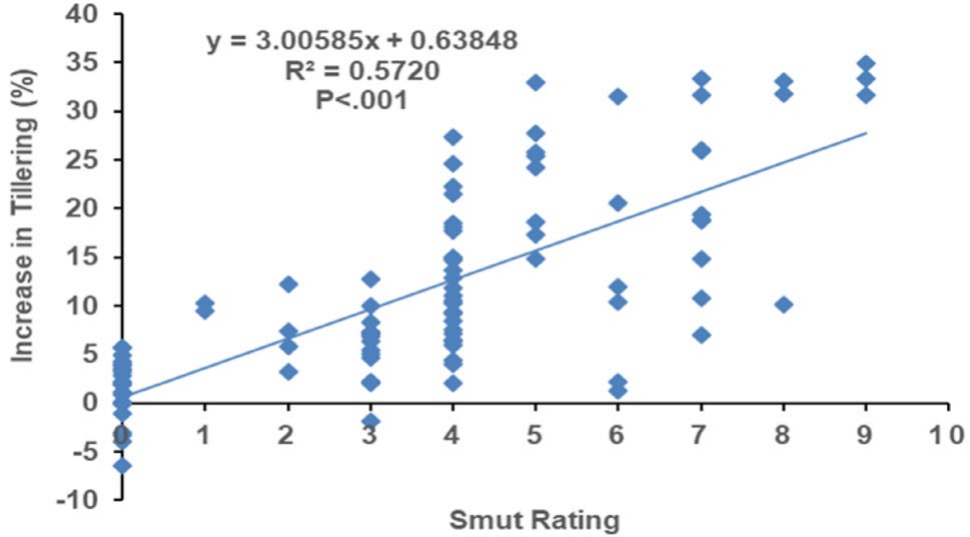
Regression analysis of tillering with the whip smut disease. Tiller numbers of all tested cultivars against each disease rating category was pooled and their mean values were used for regression analysis. Dots representing 104 cultivars artificially infested with *Sporisorium scitamineum*. Numbers of tillers originated from each plant (bud) were counted till 120 days of plantation.

The regression analysis equation (y = 3.00585x + 0.63848) for predicting the influence of smut disease rating on variation in the number of tillers in sugarcane plants shows a positive and significant (*P* < 0.001) relationship between the disease rating and tiller numbers (R^2^ = 0.5720). Additionally, it can be forecast by regression equation that with the increase in one unit/rate of disease would result in 0.63848% (regression coefficient) tillers increment (Fig. 3).

## Discussion

Whip smut is one of the most prevalent diseases in almost all sugarcane growing regions of the world. It possesses very high destructive potential and thereby causing substantial yield losses in susceptible sugarcane cultivars^26,27^. The high level of susceptibility to whip smut forced to stop the cultivation of several superior cultivars^8,10^. Although, the disease could be managed by chemical fungicides, however are very laborious, time consuming and costly, besides other disadvantages. The most reliable and economical means for smut disease management is cultivation of resistant sugarcane cultivars^21,28^. In most host–pathogen scenario, yield losses have been determined by frequency of parasitism (incidence) and injury level (severity). Mostly high values of incidence with corresponding minimal yield losses demonstrated that resistance mechanism was operated horizontally. Therefore, plants having slight susceptibility are good choice for selection as indication of horizontal nature of resistance^29^. Varietal ‘boom and bust cycle’ resulted in the loss of resistance within a few years, which happened mostly due to the development of new pathotypes in pathogen population^2,30^. Durability of resistant to smut pathogen is established. Various degrees of resistant have been observed in present study. Out of 104 sugarcane cultivars, 26 were found immune, 2 very highly resistant, 4 highly resistant, 15 resistant, 27 moderately resistant, 8 intermediate, 6 moderately susceptible, 10 susceptible, 3 highly susceptible and 3 were very highly susceptible. In recent years, several trials evaluating smut resistance have been undertaken around the world. In China, 15 sugarcane cultivars were selected for a field experiment to establish the smut resistance evaluation system^31^. In Ethiopia, 31 imported sugarcane cultivars (provided by the CIRD) were screened against Ethiopian isolates of smut pathogen, and about 81% accessions were from moderately resistant to very highly resistant reactions^32^. Resistance against smut can be obtained from wild relatives of *Sacchrum*, out of 79 accessions resulting from back crosses of *Erianthus arundinaceus* x commercial cultivars, 10 shown highly resistant to moderately resistant reaction to the whip smut^28^. The varietal response and intensity of the whip smut disease can vary from location to location^33^. In another study conducted at Faisalabad (Pakistan), it revealed that out of 15 cultivars, 8 were resistant, 6 moderately susceptible and only one found to be susceptible to the field screening against the whip smut^34^. After the first appearance of this disease in Australia during 1998 at Ord River Irrigation Area (ORIA), about 1705 clones were screened, and 75% of them appeared susceptible to smut^35,36^. At Louisiana (USA), the smut incidence ranging from 10–20% on most susceptible cultivars grew widely at different surveyed locations^37^.

Resistance in general or specific interact with various internal and external factors leads to the stimulation, repression, or even suspension of the infection. However, systemic molecular studies of pathological reaction of sugarcane towards *S. scitamineum* are unique and preliminary^38^. The smut specific primers S1 and S2 based on the intragenic spacer region of Ustilago spp. were used for the amplification of *S. scitamineum*^25^. Pathogens DNA were successfully amplified in all the cultivars except the one that shows immune reaction in a disease screening trial. It is important to note that template was prepared from the plants which were grown from inoculated setts. We speculate that pathogen remains localized, confined to or near point of entry despite of its systemic nature. Otherwise, its amplification would be successful. Early detection of pathogen in cultivars under natural condition is preferred over traditional field screening that takes longer periods of time^16^. It may be successfully employed in designing disease prediction models. However, to measure the effects of disease on plant growth and yield, conventional screening methods are desirable. Epidemiological factors including temperature, rainfall and humidity normally have great consequences and are very important for the development and spread of pathogen causing smut of sugarcane. In the present study, we did not find any correlation among smut incidences and temperature as well as rainfall. However, smut disease incidences are positively correlative with relative humidity. In contrast to this, other scientists have observed a positive correlation between whip smut incidence and temperature and a negative relevance to the relative humidity^39^. While in Pakistan, smut incidence was found positively correlated with relative humidity and negatively with temperature^34^.

Upon successful infection, *S. scitamineum* leads to many changes in colonized host, causing vast manipulations in important qualitative and quantitative parameter. Reduction in germination and enhanced profuse tillering in susceptible cultivars is the usual characteristics of the whip smut disease. During our research, *S. scitamineum* caused a highly significant reduction in germination of the susceptible cultivars. Correlation analysis also revealed that the increasing disease severity caused a significant negative impact on setts germination. Similarly, it was also concluded that *S. scitamineum* has the potential to diminish the plant germination in the absence of any control mechanism^40^. Enhanced tillering in affected plants and susceptible cultivars was reported^41,42^. We also observed that susceptible cultivars produced more tillers as compared to resistant ones. Accordingly, we found no significant difference for tillers/plant in inoculated v/s un-inoculated plots, in those cultivars showed immune reaction. The host plant interaction is a complex phenomenon and not yet completely understood. The production of high numbers of tillers is the result of *S. scitamineum* action on infected canes due to termination of their apical growth, thus creating profuse vegetative growth from the base to compensate for the loss^43^. The poor cane formation coupled with profuse tillering, gives “grassy” appearance to affected plants of highly susceptible cultivars^44^.

## Conclusions

In terms of smut susceptibility, a wide range of diversity exists in 104 evaluated germplasms, which would be utilized in sugarcane production or in breeding programs. In resistance field screening, the maximum smut incidence of 54.10% was recorded on a susceptible cultivar CP-29-120. The result from PCR detection on pathogen is in confirmation with that of field screening trials, but limited to distinguish the germplasm into two groups only i.e., immune and not immune. Pathogen infection not only caused remarkable reduction in setts germination, but also adversely affected the tillering in susceptible cultivars. Correlation analysis demonstrated that the increasing disease severity caused a significant (*P* < 0.001) negative impact on setts germination. And the regression analysis showed a positive and significant (*P* < 0.001) relationship between the disease rating and tiller numbers (R^2^ = 0.5720). These results obtained here should be helpful for the evaluation of smut resistance in sugarcane germplasm, and it also screened out several excellent germplasms for potential application in sugarcane breeding.

### Supplementary Information

Supplementary Figure 1.

Supplementary Tables.

## Data Availability

The datasets analysed during this study are included in this manuscript and its supplementary file.
