# Supplementary material for: Screening of sugarcane germplasm against Sporisorium scitamineum and its effects on setts germination and tillering
Source: Sci Rep. 2024 Jun 25;14:14653. doi: 10.1038/s41598-024-64810-1 (PMC11199591; doi:10.1038/s41598-024-64810-1)
Supplement: Supplementary file 1 — Supplementary Figure 1. [file 41598_2024_64810_MOESM1_ESM.docx]

*
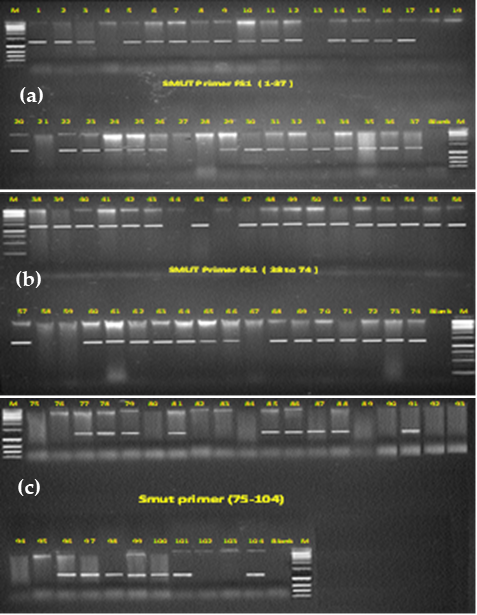
*

**Supplementary figure 1.** Analysis of 104 sugarcane cultivars against Sporisorium scitamineum by PCR amplification with smut primer FS1.

Where; **(a)** M=DNA marker, 1=Chandka, 2=Q-88, 3=Larkana-2001, 4=S-2006-SP-30, 5=S-2006-SP-18, 6=HoTh-409, 7=CPF-229, 8=Co-208, 9=H-86-NSG-311, 10=CP-85-SP-571, 11=Th-725, 12=CPS-1827, 13=BPTh-807, 14=SPSG-3481, 15=S-2003-CPSG-704, 16=S-2002-HSG-200, 17=CPSG-244-S-2083, 18=CP-70-530, 19=S-2003-QSSG-776, 20=CSSG-2402, 21=S-2003-US-633, 22=CP-59-1059, 23=HoTh-550, 24=CP-29-120, 25=S-2003-US-160, 26=HSF-240, 27=QSG-1741, 28=HoTh-516, 29=Th-702, 30=NCo-310, 31=CP-82-2083, 32=CSSG-1741, 33=S-2003-HoSG-701, 34=HoTh-424, 35=HoTh-316, 36=BPTh-804, 37=HoTh-408, B=blank.

**(b)** M=DNA marker, 38=HoTh-513, 39=S-2003-US-704, 40=HoTh-517, 41=HoTh-419, 42=HoTh-518, 43=HoTh-432, 44=HoTh-438, 45=Co-639, 46=CB-2919, 47=S-2003-HoSG-1626, 48=CoJ-84, 49=CP-52-28, 50=HoTh-401, 51=CP-70-SP- 1215, 52=HoTh-326, 53=S-2003-CPSG-193, 54=Th-720, 55=NSG-60, 56=Co-620, 57=CP-69-1059, 58=HoTh-544, 59=Th-704, 60=CPD-01-359, 61=CSSG-2476, 62=B-46364, 63=HoTh-127, 64=S-2003-HoSG-679, 65=HoTh-518, 66=CoJ-81, 67=HoTh-318, 68=B-43405, 69=Co-413, 70=HoTh-612, 71=CP-75-1353, 72=Triton, 73=Co-1148, 74=HoTh-344, B=blank.

**(c)** M=DNA marker, 75=HoTh-610, 76=HoTh-4140, 77=YT-236, 78=S-2002-SFSD-1307, 79=Th-10, 80=Roc-16, 81=AP-98-156/01, 82=AP-98-156/02, 83=AP-98-156/03, 84=AP-98-156/04, 85=AP-98-156/05, 86=AP-98-156/06, 87=AP-04-59/01, 88=AP-04-59/02, 89=AP-04-68/01, 90=AP-98-156/07, 91=AP-98-156/08, 92=AP-98-103/01, 93=AP-97-69/01, 94=AP-97-56/02, 95=AP-97-56/03, 96=AP-04-46/02, 97=AP-04-46/03, 98=AP-04-59/03, 99=AP-04-68/02, 100=AP-04-68/03, 101=BP-TJ-15/01, 102=BP-TJ-651/18, 103=BP-TJ-651/20, 104=S-2006-SP-658, B=blank.

Sugarcane smut has been found in Papua New Guinea in 2016, and report in Tom et al. 2017
