# Supplementary material for: Screening of sugarcane germplasm against Sporisorium scitamineum and its effects on setts germination and tillering
Source: Sci Rep. 2024 Jun 25;14:14653. doi: 10.1038/s41598-024-64810-1 (PMC11199591; doi:10.1038/s41598-024-64810-1)
Supplement: Supplementary file 2 — Supplementary Tables. [file 41598_2024_64810_MOESM2_ESM.docx]

Supplementary table 1. List of cultivars used for screening against the whip smut caused by *Sporisorium scitamineum*

| **S. No** | **Cultivars** | **Source*** | **S. No** | **Cultivars** | **Source*** |
| --- | --- | --- | --- | --- | --- |
| 1 | HoTh-409 | NSTHRI,  Thatta | 53 | NIA-2004 | NIA, Tandojam |
| 2 | Th-725 |  | 54 | Chandka | QAARI, Larkana |
| 3 | BPTh-807 |  | 55 | Larkana-2001 |  |
| 4 | HoTh-550 |  | 56 | S-2006-SP-30 | AARI, Faisalabad |
| 5 | HoTh-516 |  | 57 | S-2006-SP-18 |  |
| 6 | Th-702 |  | 58 | CPF-229 |  |
| 7 | HoTh-424 |  | 59 | CP-85- SP-571 |  |
| 8 | HoTh-316 |  | 60 | S-2003-US-633 |  |
| 9 | BPTh-804 |  | 61 | S-2003-US-160 |  |
| 10 | HoTh-408 |  | 62 | HSF-240 |  |
| 11 | HoTh-513 |  | 63 | S-2003-US-704 |  |
| 12 | HoTh-517 |  | 64 | CP-70-SP-1215 |  |
| 13 | HoTh-419 |  | 65 | S-2002-SFSD-1307 |  |
| 14 | HoTh-518 |  | 66 | CPF-134 |  |
| 15 | HoTh-432 |  | 67 | Co-208 | SBRI, Coimbatore,  India |
| 16 | HoTh-401 |  | 68 | NCo-310 |  |
| 17 | HoTh-326 |  | 69 | Co-639 |  |
| 18 | Th- 720 |  | 70 | Co-620 |  |
| 19 | HoTh-544 |  | 71 | Co-413 |  |
| 20 | Th-704 |  | 72 | Co-1148 |  |
| 21 | HoTh-127 |  | 73 | H-86-NSG–311 | SSRI, Jhang |
| 22 | HoTh-518 |  | 74 | SPSG-3481 |  |
| 23 | HoTh-612 |  | 75 | S-2003-CPSG-704 |  |
| 24 | HoTh-344 |  | 76 | S-2002-HSG -200 |  |
| 25 | HoTh-610 |  | 77 | CPSG-244-S-2083 |  |
| 26 | HoTh-4140 |  | 78 | S-2003-QSSG -776 |  |
| 27 | Th-10 |  | 79 | CSSG-2402 |  |
| 28 | Q- 88 | ARI,  Tandojam | 80 | QSG-1741 |  |
| 29 | AP-98-156/01 |  | 81 | CSSG-1741 |  |
| 30 | AP-98-156/02 |  | 82 | S-2003-HoSG-701 |  |
| 31 | AP-98-156/03 |  | 83 | S-2003-HoSG-1626 |  |
| 32 | AP-98-156/04 |  | 84 | CoJ-84 |  |
| 33 | AP-98-156/05 |  | 85 | S-2003-CPSG-193 |  |
| 34 | AP-98-156/06 |  | 86 | NSG-60 |  |
| 35 | AP-04-59/01 |  | 87 | CSSG-2476 |  |
| 36 | AP-04-59/02 |  | 88 | S-2003-HoSG-679 |  |
| 37 | AP-04-68/01 |  | 89 | CoJ-81 |  |
| 38 | AP-98-156/07 |  | 90 | SPSG-26 |  |
| 39 | AP-98-156/08 |  | 91 | YT-236 | GARI, China |
| 40 | AP-98-103/01 |  | 92 | Roc-16 |  |
| 41 | AP-97-69/ 01 |  | 93 | CPS-1827 | SCRI, Mardan |
| 42 | AP-97-56/02 |  | 94 | CP-70-530 |  |
| 43 | AP-97-56/03 |  | 95 | CP-59-1059 |  |
| 44 | AP-04-46/02 |  | 96 | CP-29-120 |  |
| 45 | AP-04-46/03 |  | 97 | CP-82-2083 |  |
| 46 | AP-04-59/03 |  | 98 | CP-52-28 |  |
| 47 | AP-04-68/02 |  | 99 | CP-69-1059 |  |
| 48 | AP-04-68/03 |  | 100 | CP-75-1353 |  |
| 49 | BP-TJ-15/01 |  | 101 | Tritan |  |
| 50 | BP-TJ-651/18 |  | 102 | CB-2919 | Campos, Brazil |
| 51 | BP-TJ-651/20 |  | 103 | B-43405 | Barbados |
| 52 | NIA-98 | NIA,  Tandojam | 104 | B-46364 |  |

* NSTHRI: National Sugar and Tropical Horticulture Research Institute, Thatta, Pakistan, ARI: Agriculture Research Institute, Tandojam, Pakistan, NIA: Nuclear Institute of Agriculture, Tandojam, Pakistan, QAARI: Quaid-e-Awam Agricultural Research Institute, Larkana, Pakistan, SRI, AARI: Ayub Agricultural Research Institute, Faisalabad, Pakistan, SSRI: Shakarganj Sugar Research Institute, Jhang, Pakistan, SCRI: Sugar Crops Research Institute, Mardan, Pakistan, SBRI: Sugarcane Breeding Research Institute, Coimbatore, India, GARI: Guangzhou Agricultural Research Institute, Guangzhou, China.

Supplementary table 2**.** Disease incidence of the whip smut and reaction of sugarcane cultivars in the field trial, artificially inoculated with Sporisorium scitamineum.

| **S.No.** | **Cultivars** | **Disease incidence (%)** | **Disease rating /Host reaction** | **DNA amplification** |
| --- | --- | --- | --- | --- |
| 1 | BPTh-807 | 0.00 D | Immune | _ |
| 2 | BP-TJ-651/18 | 0.00 D | Immune | _ |
| 3 | BP-TJ-651/20 | 0.00 D | Immune | _ |
| 4 | CB-2919 | 0.00 D | Immune | _ |
| 5 | CP-70-530 | 0.00 D | Immune | _ |
| 6 | HoTh-318 | 0.00 D | Immune | _ |
| 7 | HoTh-4140 | 0.00 D | Immune | _ |
| 8 | HoTh-438 | 0.00 D | Immune | _ |
| 9 | HoTh-516 | 0.00 D | Immune | _ |
| 10 | HoTh-544 | 0.00 D | Immune | _ |
| 11 | HoTh-610 | 0.00 D | Immune | _ |
| 12 | QSG-1741 | 0.00 D | Immune | _ |
| 13 | Roc-16 | 0.00 D | Immune | _ |
| 14 | S-2003-QSSG -776 | 0.00 D | Immune | _ |
| 15 | S-2003-US-633 | 0.00 D | Immune | _ |
| 16 | S-2006-SP-30 | 0.00 D | Immune | _ |
| 17 | Th-704 | 0.00 D | Immune | _ |
| 18 | AP-98-103/01 | 0.00 D | Immune | _ |
| 19 | AP-98-156/02 | 0.00 D | Immune | _ |
| 20 | AP-98-156/03 | 0.00 D | Immune | _ |
| 21 | AP-98-156/04 | 0.00 D | Immune | _ |
| 22 | AP-98-156/07 | 0.00 D | Immune | _ |
| 23 | AP-97-56/02 | 0.00 D | Immune | _ |
| 24 | AP-97-56/03 | 0.00 D | Immune | _ |
| 25 | AP-04-68/01 | 0.00 D | Immune | _ |
| 26 | AP-97-69/ 01 | 0.00 D | Immune | _ |
| 27 | HoTh-344 | 1.53± 0.97 cd | VHR | + |
| 28 | AP-04-46/03 | 2.31± 1.03 b-d | VHR | + |
| 29 | CP-82-2083 | 4.28± 0.87 z, A-D | HR | + |
| 30 | HoTh-518 | 3.27± 2.08 a-d | HR | + |
| 31 | S-2002-HSG-200 | 4.93 ± 0.12 yz, A-C | HR | + |
| 32 | AP-98-156/06 | 4.77± 0.73 yz, A-C | HR | + |
| 33 | BPTh-804 | 6.25± 1.05 w-z, A-C | R | + |
| 34 | CPS-1827 | 6.56± 0.90 w-z, AB | R | + |
| 35 | Chandka | 6.01± 0.86 w-z, A-C | R | + |
| 36 | Co-620 | 7.27± 1.34 u-z, A | R | + |
| 37 | CPSG-244-S-2083 | 6.50± 1.05 w-z, AB | R | + |
| 38 | HoTh-419 | 6.64± 1.15 w-z, AB | R | + |
| 39 | HoTh-424 | 7.08± 1.17 u-z, A | R | + |
| 40 | HoTh-513 | 6.59± 1.32 w-z, AB | R | + |
| 41 | HoTh-517 | 5.60± 0.81 x-z, A-C | R | + |
| 42 | S-2003-HoSG-679 | 6.66± 1.24 v-z, AB | R | + |
| 43 | S-2003-US-160 | 6.18± 0.94 w-z, A-C | R | + |
| 44 | Th-720 | 6.74± 1.64 u-z, AB | R | + |
| 45 | AP-04-59/02 | 5.65± 0.66 x-z, A-C | R | + |
| 46 | AP-04-59/03 | 6.68± 1.14 v-z, AB | R | + |
| 47 | AP-04-68/03 | 6.29± 1.10 w-z, A-C | R | + |
| 48 | B-43405 | 7.87± 1.49 s-z, A | MR | + |
| 49 | B-46364 | 8.18± 1.44 s-z | MR | + |
| 50 | CPF-229 | 9.86±1.64 p-x | MR | + |
| 51 | Co-413 | 12.51± 2.34 m-s | MR | + |
| 52 | CP-52-28 | 10.74± 1.70 o-w | MR | + |
| 53 | CP-70-SP-1215 | 8.21± 1.44 s-z | MR | + |
| 54 | CP-85-SP-571 | 9.28±1.39 r-y | MR | + |
| 55 | CSSG-2402 | 12.43± 1.46 m-t | MR | + |
| 56 | CSSG-2476 | 8.73± 4.89 s-z | MR | + |
| 57 | H-86-NSG-311 | 9.81± 1.54 p-x | MR | + |
| 58 | HoTh–316 | 7.61± 0.84 u-z, A | MR | + |
| 59 | HoTh-127 | 8.53± 1.50 s-z | MR | + |
| 60 | HoTh-326 | 7.68± 1.64 t-z, A | MR | + |
| 61 | HoTh-432 | 9.50± 1.88 q-y | MR | + |
| 62 | HoTh-518 | 8.72± 1.94 s-z | MR | + |
| 63 | HoTh-612 | 7.73± 1.64 t-z, A | MR | + |
| 64 | NSG-60 | 11.49± 1.24 n-u | MR | + |
| 65 | Q-88 | 8.15± 1.43 s-z | MR | + |
| 66 | S-2003-CPSG-704 | 10.60± 1.55 o-w | MR | + |
| 67 | S-2006-SP-18 | 10.23± 2.05 p-x | MR | + |
| 68 | S-2003-CPSG-193 | 8.91± 1.40 s-z | MR | + |
| 69 | SPSG-3481 | 9.32± 1.66 r-y | MR | + |
| 70 | Th-702 | 8.60± 1.46 s-z | MR | + |
| 71 | Th-725 | 7.98± 1.17 s-z, A | MR | + |
| 72 | Th-10 | 11.42±1.60 n-v | MR | + |
| 73 | AP-04-68/02 | 9.93± 2.27 p-x | MR | + |
| 74 | BP-TJ-15/01 | 8.47± 1.59 s-z | MR | + |
| 75 | CoJ-84 | 15.16± 1.39 g-o | I | + |
| 76 | CP-75-1353 | 14.26± 6.30 j-q | I | + |
| 77 | HoTh-401 | 13.85± 2.41 l-r | I | + |
| 78 | HSF-240 | 14.56± 2.35 h-p | I | + |
| 79 | NCo-310 | 14.46± 1.37 i-p | I | + |
| 80 | S-2003-US-704 | 15.27± 2.15 g-o | I | + |
| 81 | S-2006-SP-658 | 13.94± 1.59 k-r | I | + |
| 82 | AP-04-46/02 | 14.03± 1.73 k-r | I | + |
| 83 | Co-639 | 17.63± 1.88 f-l | MS | + |
| 84 | S-2003-HoSG-1626 | 15.85± 2.73 f-n | MS | + |
| 85 | YT-236 | 16.44± 1.91 f-m | MS | + |
| 86 | AP-98-156/05 | 15.85± 1.69 f-n | MS | + |
| 87 | AP-98-156/08 | 16.00± 2.12 f-n | MS | + |
| 88 | AP-04-59/01 | 16.09± 1.15 f-n | MS | + |
| 89 | Co-1148 | 18.48± 1.78 f-l | S | + |
| 90 | CoJ-81 | 18.23± 1.47 f-l | S | + |
| 91 | CP-59-1059 | 19.32± 2.72 fgh | S | + |
| 92 | CP-69-1059 | 18.83± 1.32 f-j | S | + |
| 93 | HoTh-408 | 20.36± 1.63 ef | S | + |
| 94 | HoTh-409 | 19.37± 1.58 fg | S | + |
| 95 | Larkana-2001 | 19.42± 1.63 fg | S | + |
| 96 | S-2002-SFSD-1307 | 19.04± 1.67 f-i | S | + |
| 97 | S-2003-HoSG-701 | 20.44± 1.61 ef | S | + |
| 98 | AP-98-156/01 | 18.68± 2.02 f-k | S | + |
| 99 | Co-208 | 25.43± 3.67 cd | HS | + |
| 100 | CPD-01-359 | 24.44± 5.06 de | HS | + |
| 101 | Tritan | 25.08± 4.35 cde | HS | + |
| 102 | CP-29-120 | 54.10± 0.70 a | VHS | + |
| 103 | CSSG-1741 | 29.48± 6.01 bc | VHS | + |
| 104 | HoTh-550 | 33.76± 3.40 b | VHS | + |

**Supplementary table. 3.** Effects of Sporisorium scitamineum on setts germination (or sprouting) of sugarcane cultivars in the field trial.

| **S.No** | **Cultivars** | **Germination Percent** | | **Reduction**  **Percent** | **t-value** |
| --- | --- | --- | --- | --- | --- |
|  |  | **Inoculated** | **Natural Infection** |  |  |
| 1 | BPTh-807 | 52.87±0.21^e-i^ | 52.51±0.46^S-W^ | -0.68 | 0.94 |
| 2 | BP-TJ-651/18 | 53.60±0.33^c-g^ | 53.80± 0.12^P-R^ | 0.38 | -0.52 |
| 3 | BP-TJ-651/20 | 53.20±0.43^d-h^ | 53.22±0.31^R-U^ | 0.03 | -0.03 |
| 4 | CB-2919 | 51.72±0.36^i-l^ | 52.54±0.33^S-V^ | 1.59 | -1.42 |
| 5 | CP-70-530 | 50.06±0.29^o-r^ | 50.74±0.0.37^Z^ | 1.22 | -1.32 |
| 6 | HoTh-318 | 52.80±0.45^f-i^ | 53.72±0.28^QR^ | 1.73 | -1.68 |
| 7 | HoTh-4140 | 52.28±0.36^h-k^ | 52.16±0.17^V-X^ | -0.24 | 0.34 |
| 8 | HoTh-438 | 54.72±0.27^c^ | 55.48±0.27^K-M^ | 1.37 | -1.75 |
| 9 | HoTh-516 | 66.22±0.36^a^ | 66.27±0.30^c^ | 0.08 | -0.08 |
| 10 | HoTh-544 | 51.25±0.18^k-o^ | 51.78±0.14^V-X^ | 1.03 | -2.05 |
| 11 | HoTh-610 | 56.63±0.38^b^ | 55.91±0.48^I-K^ | -1.28 | 1.17 |
| 12 | QSG-1741 | 53.94±0.45^c-f^ | 54.99±0.44^L-O^ | 1.91 | -2.35 |
| 13 | Roc-16 | 48.25±0.28^v-z, A-D^ | 48.81±0.17^a^ | 1.14 | -1.72 |
| 14 | S-2003-QSSG-776 | 46.50±0.76^F-K^ | 47.17±0.72^b^ | 1.43 | -1.73 |
| 15 | S-2003-US-633 | 52.52±0.48^g-j^ | 52.42±0.34^T-W^ | -0.18 | 0.20 |
| 16 | S-2006-SP-30 | 51.82±0.36^i-l^ | 51.60±0.18^W-Z^ | -0.41 | 0.81 |
| 17 | Th-704 | 51.40±0.38^j-n^ | 50.84±0.24^YZ^ | -1.09 | 1.00 |
| 18 | AP-98-103/01 | 50.59±0.59^l-p^ | 51.33±0.32^X-Z^ | 1.43 | -0.83 |
| 19 | AP-98-156/02 | 51.70±0.35^i-m^ | 51.94±0.31^V-X^ | 0.47 | -0.68 |
| 20 | AP-98-156/03 | 54.35±0.45^cd^ | 55.09±0.49^K-N^ | 1.63 | -1.29 |
| 21 | AP-98-156/04 | 54.79±0.54^c^ | 54.58±0.55^M-Q^ | -0.39 | 0.45 |
| 22 | AP-98-156/07 | 56.78±0.15^b^ | 56.80±0.46^HI^ | 0.02 | -0.02 |
| 23 | AP-97-56/02 | 53.45±0.35^d-h^ | 54.11±0.44^O-R^ | 1.22 | -0.98 |
| 24 | AP-97-56/03 | 52.21±0.35^h-k^ | 52.33±0.23^U-W^ | 0.23 | -0.26 |
| 25 | AP-04-68/01 | 54.04±0.41^c-f^ | 54.83±0.44^M-O^ | 1.45 | -1.19 |
| 26 | AP-97-69/ 01 | 52.34±0.37^h-k^ | 53.25±0.43^R-T^ | 1.71 | -1.36 |
| 27 | HoTh-344 | 48.05±0.28^x-z,A-D^ | 62.89±0.33^i-k^ | 23.59 | -25.47^**^ |
| 28 | AP-04-46/03 | 47.42±0.46^A-G^ | 59.37±0.33^w-z,A^ | 20.13 | -23.09^**^ |
| 29 | CP-82-2083 | 44.89±0.52^L-P^ | 60.68±0.37^p-t^ | 26.08 | -29.86^**^ |
| 30 | HoTh-518 | 45.89±0.36^I-M^ | 60.50±0.34^q-u^ | 24.14 | -28.19^**^ |
| 31 | S-2002-HSG-200 | 44.92±0.56^L-P^ | 59.59±0.29^u-z^ | 24.63 | -28.68^**^ |
| 32 | AP-98-156/06 | 54.32±0.71^cd^ | 67.57±0.20^b^ | 19.45 | -21.48^**^ |
| 33 | BPTh-804 | 47.54±0.69^z,A-G^ | 63.48±0.37^g-i^ | 25.10 | -28.69^**^ |
| 34 | CPS-1827 | 57.28±0.49^b^ | 64.75±0.32^d-f^ | 11.54 | -12.03^**^ |
| 35 | Chandka | 49.48±0.39^p-v^ | 56.79±0.29^HI^ | 12.87 | -13.08^**^ |
| 36 | Co-620 | 41.62±0.28^S-U^ | 55.77±0.25^J-L^ | 25.37 | -29.06^**^ |
| 37 | CPSG-244-S-2083 | 51.64±0.67^i-n^ | 62.85±0.31^i-k^ | 17.83 | -19.39^**^ |
| 38 | HoTh-419 | 45.52±0.25^K-O^ | 58.74±0.42^z,A-D^ | 22.51 | -25.05^**^ |
| 39 | HoTh-424 | 47.61±0.51^yz,A-G^ | 60.11±0.24^s-x^ | 20.80 | -23.24^**^ |
| 40 | HoTh-513 | 46.39±0.64^G-K^ | 59.07±0.30^yz,AB^ | 21.47 | -23.93^**^ |
| 41 | HoTh-517 | 46.38±0.62^G-K^ | 60.99±0.36^n-s^ | 23.96 | -26.20^**^ |
| 42 | S-2003-HoSG-679 | 45.54±0.31^K-O^ | 60.69±0.34^p-t^ | 24.96 | -28.77^**^ |
| 43 | S-2003-US-160 | 47.68±0.44^yz,A-F^ | 60.45±0.35^q-u^ | 20.87 | -23.83^**^ |
| 44 | Th-720 | 49.92±0.42^p-s^ | 64.03±0.32^fg^ | 22.03 | -24.78^**^ |
| 45 | AP-04-59/02 | 51.18±0.37 ^k-o^ | 58.31±0.33^B-D^ | 12.23 | -13.19^**^ |
| 46 | AP-04-59/03 | 43.94±0.28^PQ^ | 58.97±0.26^y-z,A-C^ | 25.48 | -29.49^**^ |
| 47 | AP-04-68/03 | 47.82±0.58^x-z,A-E^ | 59.24±0.33^x-z,A^ | 19.27 | -20.96^**^ |
| 48 | B-43405 | 46.50±0.37^F-K^ | 57.98±0.25^DE^ | 20.09 | -21.97^**^ |
| 49 | B-46364 | 42.15±0.30^R-U^ | 56.54±0.24^H-J^ | 25.46 | -29.35^**^ |
| 50 | CPF-229 | 46.37±0.34^G-K^ | 51.74±0.27^V-Y^ | 10.38 | -11.78^**^ |
| 51 | Co-413 | 47.36±0.34^B-G^ | 58.09±0.28^C-E^ | 18.47 | -20.53^**^ |
| 52 | CP-52-28 | 45.98±0.15^H-L^ | 61.28±0.41^m-q^ | 24.96 | -28.77^**^ |
| 53 | CP-70-SP-1215 | 49.69±0.25^p-t^ | 60.98±0.35^n-s^ | 17.97 | -19.42^**^ |
| 54 | CP-85- SP-571 | 51.73±0.33^i-l^ | 61.49±0.42^l-p^ | 15.86 | -17.51^**^ |
| 55 | CSSG-2402 | 43.06±0.36^qr^ | 55.09±0.24^K-N^ | 21.84 | -24.59^**^ |
| 56 | CSSG-2476 | 45.58±0.64^J-O^ | 55.87±0.28^J-L^ | 18.42 | -20.51^**^ |
| 57 | H-86-NSG-311 | 49.33±0.52^q-w^ | 62.29±0.43^j-l^ | 20.80 | -23.23^**^ |
| 58 | HoTh-316 | 47.89±0.58^x-z,A-E^ | 59.47±0.27^v-z^ | 19.45 | -21.49^**^ |
| 59 | HoTh-127 | 47.56±0.40^z,A-G^ | 58.73±0.37^z,A-D^ | 19.02 | -20.33^**^ |
| 60 | HoTh-326 | 48.44±0.29^u-z,A-C^ | 63.02±0.46^ij^ | 23.14 | -25.12^**^ |
| 61 | HoTh-432 | 47.38±0.36^A-G^ | 63.08±0.22^h-j^ | 24.82 | -28.76^**^ |
| 62 | HoTh-518 | 47.20±0.30^,C-H^ | 59.86±0.28^t-y^ | 21.14 | -23.55^**^ |
| 63 | HoTh-612 | 42.42±0.64^R-T^ | 52.48±0.36^S-W^ | 19.17 | -20.66^**^ |
| 64 | NSG-60 | 45.35±0.38^K-O^ | 57.83±0.23^D-G^ | 21.57 | -24.22^**^ |
| 65 | Q-88 | 51.18±0.47^k-o^ | 67.48±0.21^b^ | 24.16 | -26.64^**^ |
| 66 | S-2003-CPSG-704 | 49.34±0.27^q-w^ | 57.95±0.28^D-F^ | 14.86 | -16.08^**^ |
| 67 | S-2006-SP-18 | 45.28±0.56^K-O^ | 60.92±0.36^o-s^ | 25.66 | -29.99^**^ |
| 68 | S-2003-CPSG-193 | 45.11±0.23^L-P^ | 57.04±0.34^F-H^ | 20.93 | -23.47^**^ |
| 69 | SPSG-3481 | 47.93±0.43^x-z,A-E^ | 56.59±0.16^H-J^ | 15.30 | -17.34^**^ |
| 70 | Th-702 | 47.85±0.31^x-z,A-E^ | 58.29±0.29^B-D^ | 17.92 | -19.33^**^ |
| 71 | Th-725 | 50.43±0.78^n-q^ | 61.04±0.49^n-r^ | 17.39 | -19.07^**^ |
| 72 | Th-10 | 42.66±0.45^RS^ | 53.27±0.36^R-T^ | 19.82 | -21.29^**^ |
| 73 | AP-04-68/02 | 49.44±0.41^p-v^ | 61.87±0.31^l-n^ | 20.01 | -21.80^**^ |
| 74 | BP-TJ-15/01 | 53.71±0.52^c-g^ | 65.49±0.34^c-e^ | 17.98 | -19.52^**^ |
| 75 | CoJ-84 | 44.73±0.45^M-P^ | 55.81±0.38^J-L^ | 19.85 | -21.61^**^ |
| 76 | CP-75-1353 | 48.83±0.58^r-y^ | 61.99±0.38^k-m^ | 21.23 | -23.62^**^ |
| 77 | HoTh-401 | 48.53±0.43^t-z,AB^ | 59.87±0.34^t-y^ | 18.97 | -20.18^**^ |
| 78 | HSF-240 | 46.79±0.69^z,E-J^ | 60.36±0.23^r-v^ | 22.48 | -24.93^**^ |
| 79 | NCo-310 | 45.51±0.29^K-O^ | 58.94±0.28^z,A-C^ | 22.78 | -25.65^**^ |
| 80 | S-2003-US-704 | 49.01±0.72^r-x^ | 60.84±0.39^o-s^ | 19.45 | -21.49^**^ |
| 81 | S-2006-SP-658 | 51.45±0.38^j-n^ | 61.74±0.30^l-o^ | 16.68 | -18.81^**^ |
| 82 | AP-04-46/02 | 41.84±0.46^R-U^ | 53.37±0.29^RS^ | 21.60 | -24.30^**^ |
| 83 | Co-639 | 45.10±0.64^L-P^ | 55.05±0.21^K-N^ | 18.08 | -20.09^**^ |
| 84 | S-2003-HoSG-1626 | 44.87±0.42^L-P^ | 60.23±0.27^r-w^ | 24.40 | -28.43^**^ |
| 85 | YT-236 | 46.03±0.51^H-L^ | 57.26±0.39^,E-H^ | 19.38 | -21.17^**^ |
| 86 | AP-98-156/05 | 57.10±0.67^b^ | 69.92±0.32^a^ | 18.33 | -20.39^**^ |
| 87 | AP-98-156/08 | 48.15±0.52^w-z,A-D^ | 60.83±0.35^o-s^ | 20.85 | -23.49^**^ |
| 88 | AP-04-59/01 | 48.62±0.40^t-z,A^ | 60.91±0.35^o-s^ | 20.18 | -22.30^**^ |
| 89 | Co-1148 | 39.32±0.28^V^ | 49.34±0.26^a^ | 20.32 | -22.60^**^ |
| 90 | CoJ-81 | 49.59±0.73^p-u^ | 64.60±0.24^ef^ | 23.24 | -25.12^**^ |
| 91 | CP-59-1059 | 46.44±0.73^G-K^ | 59.04±0.37^yz,AB^ | 21.31 | -23.61^**^ |
| 92 | CP-69-1059 | 48.70±0.34^s-z^ | 63.99±0.34^f-h^ | 23.89 | -25.90^**^ |
| 93 | HoTh-408 | 47.12±0.42^D-I^ | 55.42±0.34^K-M^ | 14.98 | -17.12^**^ |
| 94 | HoTh-409 | 50.46±0.65^m-q^ | 61.56±0.29^l-p^ | 18.26 | -20.30^**^ |
| 95 | Larkana-2001 | 49.69±0.75^p-t^ | 65.85±0.35^c^ | 24.55 | -28.35^**^ |
| 96 | S-2002-SFSD-1307 | 50.79±0.40^l-p^ | 58.53±0.31^A-D^ | 13.22 | -14.72^**^ |
| 97 | S-2003-HoSG-701 | 45.11±0.31^L-P^ | 54.64±0.26^M-P^ | 17.45 | -19.25^**^ |
| 98 | AP-98-156/01 | 54.07±0.52^c-e^ | 62.89±0.27^i-k^ | 14.01 | -15.86^**^ |
| 99 | Co-208 | 48.51±0.38^t-z,AB^ | 65.53±0.31^cd^ | 25.96 | -30.49^**^ |
| 100 | CPD-01-359 | 45.66±0.52^J-N^ | 57.00±0.32^GH^ | 19.89 | -21.72^**^ |
| 101 | Tritan | 44.38±0.62^OP^ | 57.29±0.30^E-H^ | 22.53 | -25.11^**^ |
| 102 | CP-29-120 | 44.62±0.29^N-P^ | 58.31±0.31^B-D^ | 23.49 | -25.04^**^ |
| 103 | CSSG-1741 | 40.99±0.25^U^ | 54.45±0.22^N-Q^ | 24.73 | -28.73^**^ |
| 104 | HoTh-550 | 41.26±0.33^TU^ | 49.01±0.29^a^ | 15.83 | -17.32^**^ |
|  | F-Statistics at df = 103 | 88.86 | 198.12 |  |  |
|  | LSD 0.05 | 1.2437 | 0.9149 |  |  |

Note: t-value without any asterisk sign are non-significant at 0.05, * = Significant at 0.05; and ** = Highly significant at 0.01 level; Means followed by the same letter(s) in the same column are not significantly different at 0.05 LSD.

Supplementary table. 4. Effects of whip smut *S. scitamineum* on tillers/plant of sugarcane cultivars in the field screening trial with artificial inoculation.

| **S.NO** | **Cultivars** | **Tillers/plant Percent** | | **Increase**  **Percent** | **t- value** |
| --- | --- | --- | --- | --- | --- |
|  |  | **Inoculated** | **Natural Infection** |  |  |
| 1 | BPTh-807 | 5.66±0.19^n-u^ | 5.55±0.16^e-i^ | 1.94 | 0.67 |
| 2 | BP-TJ-651/18 | 5.39±0.16^s-y^ | 5.39±0.22^f-k^ | 0.00 | 0.00 |
| 3 | BP-TJ-651/20 | 5.05±0.18^w-z,AB^ | 5.22±0.07^i-n^ | -3.33 | -0.87 |
| 4 | CB-2919 | 5.94±0.16^j-q^ | 5.83±0.17^a-e^ | 1.91 | 0.47 |
| 5 | CP-70-530 | 5.89±0.16^j-r^ | 5.55±0.16^e-i^ | 5.66 | 1.07 |
| 6 | HoTh-318 | 5.00±0.15^x-z,A-C^ | 5.05±0.16^k-q^ | -1.10 | -0.27 |
| 7 | HoTh-4140 | 5.89±0.14^j-r^ | 5.55±0.19^e-i^ | 5.66 | 1.12 |
| 8 | HoTh-438 | 4.83±0.25^z,A-E^ | 4.83±0.22^o-u^ | -0.03 | -0.01 |
| 9 | HoTh-516 | 5.55±0.14^p-v^ | 5.72±0.20^c-f^ | -3.00 | -0.59 |
| 10 | HoTh-544 | 5.39±0.16^s-y^ | 5.39±0.10^f-k^ | 0.03 | 0.01 |
| 11 | HoTh-610 | 5.73±0.08^l-t^ | 5.50±0.11^e-j^ | 4.02 | 2.17 |
| 12 | QSG-1741 | 5.51±0.06^p-w^ | 5.28±0.13^h-m^ | 4.29 | 1.45 |
| 13 | Roc-16 | 6.28±0.10^g-j^ | 6.05±0.10^a-c^ | 3.53 | 1.99 |
| 14 | S-2003-QSSG-776 | 6.00±0.19^j-p^ | 5.83±0.11^a-e^ | 2.81 | 1.00 |
| 15 | S-2003-US-633 | 5.38±0.08^s-y^ | 5.19±0.08^i-o^ | 3.41 | 1.41 |
| 16 | S-2006-SP-30 | 4.94±0.13^yz,A-D^ | 4.83±0.17^o-u^ | 2.19 | 0.49 |
| 17 | Th-704 | 5.33±0.19^s-y^ | 5.16±0.17^j-o^ | 3.16 | 0.66 |
| 18 | AP-98-103/01 | 5.17±0.17^v-z,A^ | 5.50±0.11^e-j^ | -6.39 | -2.14 |
| 19 | AP-98-156/02 | 4.61±0.16^B-E^ | 4.39±0.10^v-z,A^ | 4.85 | 1.09 |
| 20 | AP-98-156/03 | 6.05±0.16^i-o^ | 6.05±0.16^a-c^ | -0.03 | -0.01 |
| 21 | AP-98-156/04 | 6.16±0.24^h-m^ | 6.11±0.20^ab^ | 0.89 | 0.13 |
| 22 | AP-98-156/07 | 5.61±0.10^o-v^ | 5.39±0.20^f-k^ | 3.89 | 1.07 |
| 23 | AP-97-56/02 | 5.61±0.16^o-v^ | 5.50±0.17^e-j^ | 1.99 | 0.54 |
| 24 | AP-97-56/03 | 5.61±0.28^o-v^ | 5.83±0.08^a-e^ | -3.98 | -0.79 |
| 25 | AP-04-68/01 | 5.00±0.19^x-z,A-C^ | 4.94±0.20^m-s^ | 1.10 | 0.22 |
| 26 | AP-97-69/ 01 | 5.72±0.20^m-t^ | 5.72±0.18^c-f^ | -0.03 | -0.01 |
| 27 | HoTh-344 | 5.33±0.07^s-y^ | 4.83±0.12 ^o-u^ | 9.44 | 3.38^*^ |
| 28 | AP-04-46/03 | 5.94±0.18^j-q^ | 5.33±0.14^g-l^ | 11.09 | 3.46^*^ |
| 29 | CP-82-2083 | 6.05±0.10^i-o^ | 5.61±0.10^d-h^ | 7.38 | 2.94^*^ |
| 30 | HoTh-518 | 4.77±0.14^A-E^ | 4.50±0.22^u-z^ | 5.76 | 0.95 |
| 31 | S-2002-HSG-200 | 5.16±0.14^v-z,A^ | 5.00±0.15^l-r^ | 3.23 | 1.00 |
| 32 | AP-98-156/06 | 5.00±0.24^x-z,A-C^ | 4.39±0.13^v-z,A^ | 12.24 | 3.82^*^ |
| 33 | BPTh-804 | 5.16±0.14^v-z,A^ | 4.88±0.14^n-t^ | 5.42 | 1.39 |
| 34 | CPS-1827 | 5.39±0.06^s-y^ | 4.94±0.10^m-s^ | 8.26 | 3.20^*^ |
| 35 | Chandka | 6.11±0.07^i-n^ | 5.66±0.12^d-g^ | 7.29 | 2.71^*^ |
| 36 | Co-620 | 5.16±0.14^v-z,A^ | 5.05±0.13^k-q^ | 2.16 | 1.01 |
| 37 | CPSG-244-S-2083 | 6.17±0.18^h-m^ | 5.72±0.16^c-f^ | 7.25 | 2.67^*^ |
| 38 | HoTh-419 | 5.28±0.10^t-z^ | 4.94±0.20^m-s^ | 6.32 | 1.59 |
| 39 | HoTh-424 | 5.55±0.12^p-v^ | 5.00±0.10^l-r^ | 10.02 | 3.40^*^ |
| 40 | HoTh-513 | 6.22±0.18^g-k^ | 5.77±0.07^b-e^ | 7.16 | 2.66^*^ |
| 41 | HoTh-517 | 5.50±0.22^q-w^ | 5.22±0.08^i-n^ | 5.06 | 0.97 |
| 42 | S-2003-HoSG-679 | 4.55±0.16^C-E^ | 4.46±0.15^u-z^ | 2.05 | 0.34 |
| 43 | S-2003-US-160 | 5.89±0.22^j-r^ | 5.61±0.16^d-h^ | 4.70 | 0.91 |
| 44 | Th-720 | 5.16±0.14^v-z,A^ | 5.05±0.10^k-q^ | 2.13 | 0.59 |
| 45 | AP-04-59/02 | 6.50±0.18^f-i^ | 6.05±0.10^a-c^ | 6.92 | 2.62^*^ |
| 46 | AP-04-59/03 | 6.05±0.20^i-o^ | 6.17±0.07^a^ | -1.87 | -0.61 |
| 47 | AP-04-68/03 | 4.50±0.09^DE^ | 3.94±0.10^B^ | 12.37 | 3.91^*^ |
| 48 | B-43405 | 5.16±0.14^v-z,A^ | 4.83±0.19^o-u^ | 6.42 | 2.50 |
| 49 | B-46364 | 5.05±0.26^w-z,AB^ | 4.83±0.27^o-u^ | 4.35 | 0.42 |
| 50 | CPF-229 | 5.78±0.12^k-s^ | 5.17±0.07^j-o^ | 10.56 | 3.43^*^ |
| 51 | Co-413 | 5.39±0.22^s-y^ | 5.28±0.13^h-m^ | 2.07 | 0.34 |
| 52 | CP-52-28 | 5.55±0.09^p-v^ | 4.72±0.16^q-v^ | 15.01 | 4.27^**^ |
| 53 | CP-70-SP-1215 | 5.16±0.19^v-z,A^ | 4.05±0.10^AB^ | 21.47 | 5.01^**^ |
| 54 | CP-85- SP-571 | 6.33±0.14^g-j^ | 5.16±0.18^j-o^ | 18.46 | 4.74^**^ |
| 55 | CSSG-2402 | 6.11±0.18^i-n^ | 4.61±0.16^s-x^ | 24.54 | 5.30^**^ |
| 56 | CSSG-2476 | 5.33±0.12^s-y^ | 4.39±0.17^v-z,A^ | 17.70 | 4.42^**^ |
| 57 | H-86-NSG-311 | 5.20±0.21^u-z,A^ | 4.50±0.17^u-z^ | 13.58 | 4.06^**^ |
| 58 | HoTh-316 | 5.66±0.23^n-u^ | 5.00±0.12^l-r^ | 11.77 | 3.71^*^ |
| 59 | HoTh-127 | 5.94±0.22^j-q^ | 5.05±0.10^k-q^ | 14.95 | 4.25^**^ |
| 60 | HoTh-326 | 4.72±0.13^A-E^ | 4.39±0.10^v-z,A^ | 7.06 | 2.64^*^ |
| 61 | HoTh-432 | 4.77±0.11^A-E^ | 4.28± 0.10^x-z,AB^ | 10.47 | 3.41^*^ |
| 62 | HoTh-518 | 5.61±0.23^o-v^ | 5.28±0.10^h-m^ | 5.86 | 1.12 |
| 63 | HoTh-612 | 5.33±0.14^s-y^ | 4.83±0.08^o-u^ | 9.38 | 3.14^*^ |
| 64 | NSG-60 | 6.17±0.17^h-m^ | 5.05±0.10^k-q^ | 18.03 | 4.50^**^ |
| 65 | Q-88 | 5.16±0.18^v-z,A^ | 4.78±0.11^p-u^ | 7.49 | 2.98^*^ |
| 66 | S-2003-CPSG-704 | 5.66±0.12^n-u^ | 4.83±0.11^o-u^ | 14.74 | 4.23^**^ |
| 67 | S-2006-SP-18 | 6.05±0.16^i-o^ | 5.39±0.10^f-k^ | 11.04 | 3.40^*^ |
| 68 | S-2003-CPSG-193 | 5.44±0.13^r-x^ | 4.94±0.10^m-s^ | 9.19 | 3.12^*^ |
| 69 | SPSG-3481 | 6.05±0.12^i-o^ | 5.28±0.10^h-m^ | 12.81 | 3.97^*^ |
| 70 | Th-702 | 5.89±0.17^j-r^ | 4.28±0.10^x-z,AB^ | 27.36 | 6.89^**^ |
| 71 | Th-725 | 6.00±0.12^j-p^ | 5.50±0.10^e-j^ | 8.34 | 3.25^*^ |
| 72 | Th-10 | 5.50±0.23^q-w^ | 4.28±0.10^x-z,AB^ | 22.23 | 5.18^**^ |
| 73 | AP-04-68/02 | 5.44±0.11^r-x^ | 5.22±0.11^i-n^ | 4.04 | 1.20 |
| 74 | BP-TJ-15/01 | 5.28±0.16^t-z^ | 4.94±0.10^m-s^ | 6.35 | 1.74 |
| 75 | CoJ-84 | 6.89±0.21^d-f^ | 5.22±0.11^i-n^ | 24.18 | 5.79^**^ |
| 76 | CP-75-1353 | 6.33±0.29^g-j^ | 4.72±0.13^q-v^ | 25.43 | 6.36^**^ |
| 77 | HoTh-401 | 6.00±0.16^j-p^ | 5.11±0.15^k-p^ | 14.84 | 4.32^**^ |
| 78 | HSF-240 | 6.61±0.19^e-h^ | 4.77±0.10^p-u^ | 27.77 | 7.26^**^ |
| 79 | NCo-310 | 6.66±0.19^e-g^ | 4.94±0.10^m-s^ | 25.82 | 6.50^**^ |
| 80 | S-2003-US-704 | 6.22±0.19^g-l^ | 4.17±0.07^z,AB^ | 33.00 | 10.25^**^ |
| 81 | S-2006-SP-658 | 5.39±0.18^s-y^ | 4.39±0.07^v-z,A^ | 18.54 | 4.77^**^ |
| 82 | AP-04-46/02 | 6.11±0.30^i-n^ | 5.05±0.16^k-q^ | 17.30 | 4.34^**^ |
| 83 | Co-639 | 7.05±0.20^de^ | 4.83±0.11^o-u^ | 31.50 | 7.73^**^ |
| 84 | S-2003-HoSG-1626 | 5.16±0.11^v-z,A^ | 5.05±0.13^k-q^ | 2.16 | 1.00 |
| 85 | YT-236 | 5.61±0.20^o-v^ | 4.94±0.17^m-s^ | 11.89 | 3.74^*^ |
| 86 | AP-98-156/05 | 4.39±0.16^E^ | 4.33±0.15^w-z,A^ | 1.25 | 0.54 |
| 87 | AP-98-156/08 | 4.67±0.09^B-E^ | 4.16±0.07^yz,AB^ | 10.79 | 3.51^*^ |
| 88 | AP-04-59/01 | 6.22±0.25^g-k^ | 4.94±0.10^m-s^ | 20.53 | 4.81^**^ |
| 89 | Co-1148 | 5.72±0.11^m-t^ | 4.61±0.16^s-x^ | 19.43 | 4.80^**^ |
| 90 | CoJ-81 | 6.22±0.26^g-k^ | 5.05±0.10^k-q^ | 18.73 | 4.77^**^ |
| 91 | CP-59-1059 | 7.33±0.21^cd^ | 5.94±0.16^a-d^ | 18.92 | 4.78^**^ |
| 92 | CP-69-1059 | 6.66±0.19^e-g^ | 4.94±0.10^m-s^ | 25.84 | 6.52^**^ |
| 93 | HoTh-408 | 7.83±0.28^b^ | 5.22±0.13^i-n^ | 33.32 | 10.44^**^ |
| 94 | HoTh-409 | 7.05±0.27^de^ | 5.22±0.11^i-n^ | 25.97 | 6.67^**^ |
| 95 | Larkana-2001 | 6.16±0.21^h-m^ | 5.50±0.11^e-j^ | 10.79 | 3.49^*^ |
| 96 | S-2002-SFSD-1307 | 5.61±0.10^o-v^ | 5.22±0.11^i-n^ | 6.92 | 2.60^*^ |
| 97 | S-2003-HoSG-701 | 6.66±0.20^e-g^ | 4.55±0.19^t-y^ | 31.68 | 8.19^**^ |
| 98 | AP-98-156/01 | 5.67±0.25^n-u^ | 4.83±0.11^o-u^ | 14.74 | 4.24^**^ |
| 99 | Co-208 | 6.00±0.14^j-p^ | 5.39±0.14^f-k^ | 10.17 | 3.44^*^ |
| 100 | CPD-01-359 | 7.05±0.13^de^ | 4.72±0.11^q-v^ | 33.08 | 10.32^**^ |
| 101 | Tritan | 7.00±0.23^de^ | 4.78±0.11^p-u^ | 31.75 | 7.75^**^ |
| 102 | CP-29-120 | 8.61±0.33^a^ | 5.61±0.16^d-h^ | 34.86 | 11.22^**^ |
| 103 | CSSG-1741 | 7.00±0.15^de^ | 4.66±0.14^r-w^ | 33.35 | 10.56^**^ |
| 104 | HoTh-550 | 7.72±0.28^bc^ | 5.28±0.15^h-m^ | 31.66 | 7.73^**^ |
|  | F-Statistics at df = 103 | 17.70 | 13.03 |  |  |
|  | LSD 0.05 | 0.4886 | 0.3807 |  |  |

Note: t-value without any asterisk sign are non-significant at 0.05, * = Significant at 0.05; and ** = Highly significant at 0.01 level; Means followed by the same letter(s) in the same column are not significantly different at 0.05 LSD.
